# Evolutionary Tradeoffs

**DOI:** 10.1093/emph/eou015

**Published:** 2014-04-18

**Authors:** Peter T. Ellison

**Affiliations:** Department of Human Evolutionary Biology, Harvard University

## Definition and background

In biological systems, traits are often linked in ways that prevent simultaneous optimization of all of them. The resulting ‘evolutionary tradeoffs’ reflect necessary compromises among the functions of multiple traits. Such compromises are particularly clear when energy must be allocated among competing metabolic functions. Energy allocated to growth is not available for reproduction, immune function or other energy-consuming processes [1]. An important implication of evolutionary tradeoffs for medicine is the fact that optimal health in all domains may not be possible.

## Examples in human biology and public health

**Figure 1.**
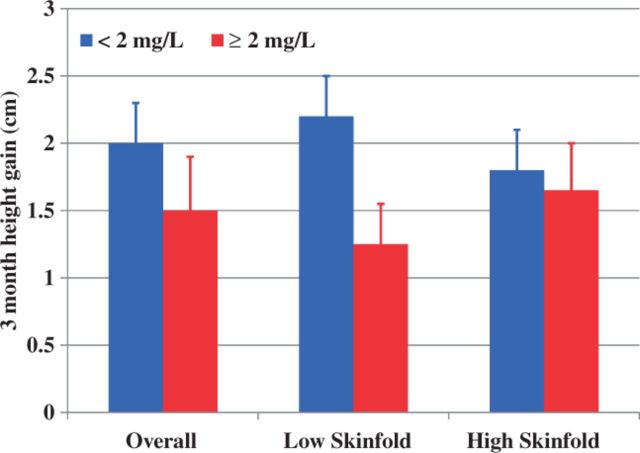
Three-month height growth (±SE) in Bolivian children with high or low/normal CRP levels and high or low skinfold measures of subcutaneous fat. Redrawn from McDade *et al.* [2].

Where energy is limiting, tradeoffs may be observed between growth and immune function. In rural Bolivia, children with elevated serum C-reactive protein (CRP), a marker of inflammation, do not gain height as rapidly over the succeeding 3 months as those with low-normal CRP levels [2]. The effect is more acute in children under 5 years of age, who are in a more rapid growth phase, than in those over five. Children with greater reserves of stored energy, as manifested by greater subcutaneous fat reserves, are relatively buffered against the tradeoff (Fig. 1) [2].

Adolescent pregnancy may result in a tradeoff between maternal and fetal growth. From the mother’s perspective, this is a tradeoff between growth and reproduction. In New Jersey, teenage girls who are still growing allocate less energy to their fetuses when pregnant than those who have completed their own growth [3]. Such a tradeoff may contribute to the elevated risk of obstetric complications in teenage pregnancies.

## Examples in clinical medicine

Prescription of hormone replacement therapy to post-menopausal women may reduce the risk of osteoporosis and ovarian cancer but increase the risk of breast cancer [4]. This tradeoff can be traced to the role of ovarian steroids as both bone trophic hormones and mitotic stimulants for target tissues in the breast. This linkage functions to help in the mobilization of calcium stores during lactation, but results in a tradeoff between bone health and cancer risk later in life.
